# Fortification and bioaccessibility of saffron apocarotenoids in potato tubers

**DOI:** 10.3389/fnut.2022.1045979

**Published:** 2022-11-30

**Authors:** Lourdes Gómez Gómez, Lucía Morote, Sarah Frusciante, José Luis Rambla, Gianfranco Diretto, Enrique Niza, Alberto José López-Jimenez, María Mondejar, Ángela Rubio-Moraga, Javier Argandoña, Silvia Presa, Alejandro Martín-Belmonte, Rafael Luján, Antonio Granell, Oussama Ahrazem

**Affiliations:** ^1^Instituto Botánico, Universidad de Castilla-La Mancha, Albacete, Spain; ^2^Departamento de Ciencia y Tecnología Agroforestal y Genética, Facultad de Farmacia, Universidad de Castilla-La Mancha, Albacete, Spain; ^3^Laboratory of Biotechnology, Italian National Agency for New Technologies, Energy, and Sustainable Development (ENEA), Casaccia Research Centre, Rome, Italy; ^4^Instituto de Biología Molecular y Celular de Plantas, Consejo Superior de Investigaciones Científicas-Universidad Politécnica de València, Valencia, Spain; ^5^Departamento de Biología, Bioquímica y Ciencias Naturales, Universitat Jaume I, Castellón de la Plana, Spain; ^6^Departamento de Ciencia y Tecnología Agroforestal y Genética, Escuela Técnica Superior de Ingenieria Agronómica y Montes, y Biotecnología, Universidad de Castilla-La Mancha, Albacete, Spain; ^7^Laboratory of Synaptic Structure, Departamento Ciencias Médicas, Facultad de Medicina, Instituto de Investigación en Discapacidades Neurológicas (IDINE), Universidad de Castilla-La Mancha, Albacete, Spain

**Keywords:** saffron, potato, crocins, picrocrocin, carotenoid cleavage dioxygenase, bioaccessibility

## Abstract

Carotenoids are C40 isoprenoids with well-established roles in photosynthesis, pollination, photoprotection, and hormone biosynthesis. The enzymatic or ROS-induced cleavage of carotenoids generates a group of compounds named apocarotenoids, with an increasing interest by virtue of their metabolic, physiological, and ecological activities. Both classes are used industrially in a variety of fields as colorants, supplements, and bio-actives. Crocins and picrocrocin, two saffron apocarotenoids, are examples of high-value pigments utilized in the food, feed, and pharmaceutical industries. In this study, a unique construct was achieved, namely O6, which contains *CsCCD2L*, *UGT74AD1*, and *UGT709G1* genes responsible for the biosynthesis of saffron apocarotenoids driven by a patatin promoter for the generation of potato tubers producing crocins and picrocrocin. Different tuber potatoes accumulated crocins and picrocrocin ranging from 19.41–360 to 105–800 μg/g DW, respectively, with crocetin, crocin 1 [(crocetin-(β-D-glucosyl)-ester)] and crocin 2 [(crocetin)-(β-D-glucosyl)-(β-D-glucosyl)-ester)] being the main compounds detected. The pattern of carotenoids and apocarotenoids were distinct between wild type and transgenic tubers and were related to changes in the expression of the pathway genes, especially from *PSY2*, *CCD1*, and *CCD4*. In addition, the engineered tubers showed higher antioxidant capacity, up to almost 4-fold more than the wild type, which is a promising sign for the potential health advantages of these lines. In order to better investigate these aspects, different cooking methods were applied, and each process displayed a significant impact on the retention of apocarotenoids. More in detail, the *in vitro* bioaccessibility of these metabolites was found to be higher in boiled potatoes (97.23%) compared to raw, baked, and fried ones (80.97, 78.96, and 76.18%, respectively). Overall, this work shows that potatoes can be engineered to accumulate saffron apocarotenoids that, when consumed, can potentially offer better health benefits. Moreover, the high bioaccessibility of these compounds revealed that potato is an excellent way to deliver crocins and picrocrocin, while also helping to improve its nutritional value.

## Introduction

Carotenoids are widely distributed isoprenoid pigments that perform important and multiple functions in all taxa (1). All photosynthetic organisms produce carotenoids since they are essential structural elements of antenna complexes and photosynthetic reaction centers and safeguard the photosynthetic apparatus against photooxidation. Carotenoids mainly accumulate in flowers and fruits, where they contribute substantially to the plant-animal communication process (2–5). In addition, these pigments are produced by many heterotrophic bacteria (6) and fungi (7). Carotenoids also confer the characteristic colorations of some crustaceans, fish, and birds (8, 9). However, animals must rely on their dietary sources to meet their carotenoid demands as they are unable to synthesis them on their own (10). By contrast, apocarotenoids are formed both in plants and in the tissues of animals that have consumed carotenoid-containing food (11). In plants, these compounds have significant roles in how plants interact with their environment, providing competitive advantages such as defense against pathogens and herbivores, chemoattractants or repellents, signals for predation and seed dispersal (12), allelochemical compounds (13), and the establishment of symbiotic associations (14). They also play a key role in plant development, acting as phytohormones, e.g., abscisic acid (ABA), mediating in biotic and abiotic stress responses, seed germination, dormancy, or leaf abscission (15); and strigolactones (SL), involved in the control of shoot branching, lateral root formation (16), and root hair elongation. In animals, apocarotenoids have the potential to control a wide range of specific cellular functions (17). In humans, the amount of beneficial health effects assigned to apocarotenoids is thus far very large and is growing even faster. These dietary carotenoid-derived compounds have been proven to have an impact on neurological disorders, cardiovascular illnesses, cancer, or metabolic syndromes (18). Thus, the health benefits attributed to carotenoids and apocarotenoids have centered more on foods and crops that contained these compounds at high levels, encouraging consumers to introduce them into their diet. Among the plants producing carotenoids and apocarotenoids with high values in the pharmaceutical industry we find saffron, also known as the golden spice, which is made from the dried stigmas of *Crocus sativus* L. Saffron’s long scarlet stigmas are highly prized for flavoring and coloring in the food industry, making it one of the most expensive spices in the world (11, 19, 20). The main components of saffron arising from the oxidative cleavage of carotenoids are *cis*- and *trans*-crocin apocarotenoids, picrocrocin (β-D-glucopyranoside of hydroxyl-β-cyclocitral), along with its degradation product, safranal (2,6,6-trimethyl-1,3-cyclohexadiene-1-carboxaldehyde) (21), whose biosynthesis and accumulation during the development of stigma has been characterized in the last decade (20, 22–25). Different carotenoid cleavage dioxygenases (CCDs) have been identified in *C. sativus* (22, 24, 26–29), with CsCCD2L being the one responsible for the generation of crocetin (26, 27). This enzyme is able to hydrolyze zeaxanthin at 7-8-7′-8′ double bonds to produce crocetin dialdehyde and two molecules of 4-hydroxy-2,6,6-trimethyl-1-cyclohexene-1-carboxaldehyde (HTCC) (11). Crocetin dialdehyde serves as a substrate for aldehyde dehydrogenase (ALDH) enzymes which render crocetin (30). This is next glycosylated by a glucosyltransferase (UGT), adding up to three glucoses to both ends of the molecule (31). The HTCC molecule is also transformed by the action of other UGTs to produce picrocrocin (32–34). Interestingly, these enzymes have also been identified and characterized in two other species, buddleja and gardenia, which are able to synthesize these apocarotenoid compounds in, respectively, their tube flowers and fruits, albeit at very low levels (33, 35, 36).

The biological effects of saffron and its prospective medical uses, notably those based on its cytotoxic, anticarcinogenic, antioxidant, and anticancer activities, have been reported (37, 38). Throughout the last decade, consumers’ preferences and interest in foods with high antioxidants have increased due to their health and nutrition properties (39). Consumption of carotenoid-rich foods may provide health benefits associated with antioxidants such as antiaging, anticancer, and anti-inflammatory properties (40). Up to now, crocins and picrocrocin production have been achieved, by transient or stable genetic transformations, in *N. benthamiana* and *N. tabacum*, and in *S. lycopersicum* (34, 41–45), but never in one of the four worldwide crops in terms of production and consumption (rice, wheat, maize, and potato).

Potato (*Solanum tuberosum* L.) is a commercially important crop throughout the world both for the fresh and the processed food industries. In addition to being the largest vegetative-propagated crop worldwide, potato tubers have become a staple in areas where there is growing pressure on limited land, and increasing food demand. Since this plant is a short season crop, it can be preserved for long periods of time, and requires minimum inputs (46). Since it is grown in a wide range of climates, the potato tuber represents an excellent plant candidate to be used as a reservoir for producing natural metabolites (47). However, while this organ has been shown to accumulate high and diversified members of phenylpropanoids in the cultivated and wild potato species (48, 49), it is devoid of carotenoids (50). Several efforts have been carried out either by metabolic engineering (51–55) or classic breeding (56, 57) in order to increase β-carotene and total carotenoid contents. On the contrary, no research on apocarotenoid profile and functions in potato is available, with the exception of the elucidation of the CCD4 role in tuber development (58). Here, we report the production of crocins and picrocrocin apocarotenoids in a heterologous plant system through the obtention of stable transgenic lines of *S. tuberosum* cv. Desirée that express the *CsCCD2L*, *UGT74AD1*, and *UGT709G1* genes under the control of the patatin promoter. Furthermore, we analyzed the content of carotenoids and apocarotenoids and the expression of genes involved in the metabolic pathway in both wild type and transgenic tubers. We also investigated the effect of different cooking procedures on the absorption of crocins. Overall, our data indicated that engineering crocins and picrocrocin biosynthesis in potato is achievable. The effects on tuber carotenoids and apocarotenoids were different between wild type (WT) and transgenic lines and were associated to changes in the expression of the biosynthetic and catabolic pathway genes. Additionally, the antioxidant activity displayed by transgenic tuber potatoes was higher than the WT, whereas the *in vitro* bioaccessibility of apocarotenoids was found to be higher in boiled potatoes as compared to raw, baked, and fried ones.

## Materials and methods

### Growth of tubers

Plants of *Solanum tuberosum* cv. Desirée were grown in 10 cm diameter compost-filled pots from seed tubers or *in vitro*-propagated tissue culture plants. Plants were grown in a greenhouse with a day/night temperature of 20/15°C. The maximum irradiance was approximately 10,500 mol m^–2^ s^–1^, and the average day length was 16 h. Plant tubers were harvested after flowering but before senescence.

### Vector construction and generation of potato transgenic plants

The Goldenbraid strategy was followed in order to construct the vectors (59, 60). Briefly, the full open reading frame (ORF) of *CsCCD2L*, *UGT74AD1*, and *UGT709G1* was synthesized using the sequence provided (26, 31, 61) by removing a *Bsm*BI and *Bsa*I restriction sites in the original sequence. The products were cloned using the Goldenbraid modular cloning system’s level 0 vector pUPD2. The plasmids pUPD2-CsCCD2L, pUPD2-UGT74AD1, and pUPD2-UGT709G1 that resulted were then used to create a recombinant binary vector as follows: O6 = pDGB32 [pPATCsCCD2LT35S-pNos-Hyg-T35S-pPATUGT74AD1T35S-pPATUGT709G1T35S-]. Plasmid was electroporated into the *Agrobacterium tumefaciens* LBA4404 strain and used for stable potato transformation into *S. tuberosum*, as previously described (62). The presence of corresponding genes in transgenic plants rooted on Hygromycin selection was confirmed using genomic DNA PCR. Positive transgenic potato plants were grown in a greenhouse after being transferred to soil. All the primers used to obtain the final constructs are listed in Supplementary Table 1.

### Apocarotenoid and carotenoid analyses

Polar and non-polar metabolites were extracted from 50 to 10 mg of lyophilized whole-tuber samples (pooled samples of three tubers), respectively. For the polar metabolite analyses, the tissue was extracted in cold 50% MeOH. The soluble fractions were analyzed using high performance liquid chromatography-diode array detector-high resolution mass spectrometry (HPLC-ESI-HRMS) and HPLC-DAD as previously described (34). A polar fractions were extracted with 1:1:2 of cold extraction solvents (MeOH:Tris:CHCl_3_), and analyzed by HPLC-APCI-HRMS and HPLC-DAD as previously described (63). Metabolites were identified using co-migration with standards, by matching the UV spectrum of each peak against that of a standard when available, based on literature data, and *m/z* accurate masses, as reported in the PubChem database^1^ for monoisotopic mass identification or using the Metabolomics Fiehn Lab Mass Spectrometry Adduct Calculator^2^ in the case of adduct detection. Pigments were quantified by integrating the peak areas which were converted to concentrations through comparison with standards and as reported previously (41).

### Transmission electron microscopy

A piece of tissue 1–2 mm thick was obtained by slicing vertically through the center of the tuber with a razor blade. The acquired slides were processed and analyzed as previously described (25).

### qRT-polymerase chain reaction analysis

For real-time quantitative reverse transcription (qRT-PCR) analysis, total RNA (2 μg) was treated with RQ1 DNase (Promega, Madrid, Spain) and reverse-transcribed using oligo dT primers, while first-strand cDNAs were synthesized by RT from 1 to 2 μg of total RNA using a first-strand cDNA synthesis kit from GE Healthcare Life Sciences (Buckinghamshire, UK) and 18mer oligo dT. Primer design was carried out using the Primer Express software. The primer used for the analyses of carotenoid and apocarotenoid was as listed (64) and (Supplementary Table 2), and Real-time PCR was achieved with a StepOne™ Thermal Cycler (Applied Biosystems, Foster City, CA, USA) and analyzed using StepOne software v2.0 (Applied Biosystems, Foster City, CA, USA). The PCR method was as follows: initial denaturation at 94°C for 5 min; 30 cycles of denaturation at 94°C for 20 s, annealing at 60°C for 20 s, and extension at 72°C for 20 s; and a final extension at 72°C for 5 min. Assays were carried out in a StepOne™ Thermal Cycler (Applied Biosystems, Foster City, CA, USA) and analyzed using the StepOne software v2.0 (Applied Biosystems, Foster City, CA, USA). Three biological and two technical replicas were performed for the expression analyses. Gene changes were determined using the 2^–ΔΔCt^ method by normalizing to the elongation factor 2 (first Δ) and then the expression was estimated for each gene by comparison to the normalized expression (second Δ).

### Volatile analyses

Volatile metabolites were trapped by means of headspace solid phase micro-extraction (HS-SPME) and analyzed by gas chromatography coupled to mass spectrometry (GC/MS) (65). Briefly, frozen tuber potato sections were ground in a cryogenic mill. Then, 500 mg of the ground material were then placed in a 15 mL glass vial and incubated at 30°C for 10 min in a water bath. Later, 2 mL of CaCl_2_ 5 M and 200 μL of an EDTA 500 mM, pH 7.5 solution were added and mixed gently. Next, 2 mL of the resulting paste was transferred to a 10 mL screw cap headspace vial with a silicon/PTFE septum. Volatile compounds were extracted from the headspace by means of a 65 μm PDMS/DVB solid phase micro-extraction fiber (SUPELCO, Madrid, Spain). The extraction of volatiles was done automatically using a CombiPAL autosampler (CTC Analytics, Zwingen, Switzerland). Vials were incubated at 50°C for 10 min with agitation at 500 rpm and, immediately, the fiber was left in contact with the vial’s headspace for 20 mins while being kept at the same temperature and agitation. Desorption was carried out at 250°C for 1 min using the splitless mode in the injection port of a 6890N gas chromatograph (Agilent Technologies, Madrid, Spain). After desorption, the fiber was cleaned for 5 min at 250°C in an SPME fiber conditioning station (CTC Analytics, Zwingen, Switzerland), under a helium flow. Chromatography was carried out on a DB-5 ms (60 m, 0.25 mm, 1.00 μm) capillary column (J&W) with helium as the carrier gas at a constant flow of 1.2 mL/min. The GC interface and MS source temperatures were 260 and 230°C, respectively. Oven programming conditions were 40°C for 2 min, 5°C/min ramp until 250°C, and a final hold at 250°C for 5 min. Data was collected in a 5975B mass spectrometer (Agilent Technologies, Madrid, Spain) in the 35–250 m/z range at 6.2 scans/s, with electronic impact ionization at 70 eV. The Enhanced ChemStation E.02.02 software was used to analyze the chromatograms (Agilent Technologies, Madrid, Spain).

### Total antioxidant activity determination by 2,2-Diphenyl-1-picrylhydrazyl assay

Free radical scavenging (FRS) activity was determined as previously described (43). Tuber flesh (50 mg lyophilized) was extracted with acetone and mixed with 0.1 mM methanolic 2,2-Diphenyl-1-picrylhydrazyl (DPPH) for 30 mins at room temperature. The absorbance of the reactions was measured at 517 nm. The FRS was computed as % = (A0 − A1/A0) 100.

### Cooking procedure and metabolite analyses

Potato tubers from WT and pooled line O6 were collected at maturity. A total of 15 visually healthy tubers were selected, rinsed with distilled water, and dried. Cooking experiments were performed in three technical replications. The tubers were cut into quarters and distributed for either raw, boiled, baked, or fried techniques. Boiling treatment was done in water in a beaker at 110°C (0.1 kg of potatoes and 250 mL of boiling water without salt), during 15 ± 2 min (from start point- introducing the tubers into the boiling water., After this time the potatoes were taken out of the water and shaken for 30 s to drain the water. The baking experiment was done in an oven at 200°C during 15 min. To perform the frying treatment, the chopped potatoes were put into a steel mesh and placed in a fryer with sunflower oil at 190.6°C for 10 min. To make sure the oil was evenly distributed, the mesh was shaken two or three times using the handle. The potatoes were removed from the fryer after 10 mins and shaken for 30 s to drain the oil. In all the cases, the treated potatoes were allowed to cool at room temperature for 5 min. Following this, the samples were ground in a coffee grinder, freeze-dried, and then stored at −80°C until further analysis.

For crocin and crocetin extraction and analyses, powder samples were weighed (0.5 ± 0.05 g), and processed as described previously (34). After extraction, aliquots of the polar and non-polar phases were immediately dried. The polar and non-polar phases were dried and examined using HPLC coupled to a diode array detector as previously reported (34). Retention time, the UV/visible light spectrum, comparison to analytical standards, and dose-response curves were used to identify and quantify apocarotenoids (41).

### *In vitro* digestion

The *in vitro* digestion was done in triplicate. For salivary digestion, the samples were treated with a 1 mL solution of α-amylase with an enzyme activity of 24.0–36.0 U/mg (70 mg/mL in phosphate buffer 0.1 M and pH 6.9) at 37°C for 2 min at 37°C at 50 rpm in a shaken water bath. After the oral phase, sample pH was adjusted to 2.5 with 0.1 M HCl and treated with 1.25 g/L of pepsin, 0.5 g/L of sodium malate, 0.5 g/L of sodium citrate, 0.42 mL/L of lactic acid, 0.5 mL/L of acetic acid, and 8.77 g/L of sodium chloride, and the volume adjusted to 50 mL. Samples were incubated in a water bath at 37°C for 60 min with slow constant stirring (130 rpm) to simulate gastric digestion conditions. After this incubation period, samples were centrifuged at 4,000 rpm for 10 min. Ten percent of the supernatant (5 mL) was extracted and stored at 4°C (labeled as gastric digests). To complete the intestinal digestion stage, the pH of the residual gastric digests was increased to 7 with NaOH 10%, and treated with 0.175 g/L of bile salt and 0.05 g/L of pancreatin, with samples incubated in a water bath at 37°C for 240 min. After centrifuging the final digests at 4,000 rpm for 10 min, the supernatants were collected and stored at −80°C for further analysis. In this study, bioaccessibility of bioactive apocarotenoids was expressed as the percentage of bioactive apocarotenoids transferred to the aqueous phase during the *in vitro* digestion (amounts of total compounds in the aqueous phase/amount compounds in the sample × 100).

## Results

The aim of this work was to produce potatoes fortified in crocin, picrocrocin, and safranal—compounds that are well known for giving saffron its distinctive color, flavor, and fragrance (Supplementary Figure 1). Furthermore, these apocarotenoids show bioactive properties with several therapeutical and pharmacological applications (66). We also aimed to determine the impact of these apocarotenoids on antioxidant activities, along with their *in vitro* bioaccessibility in raw, boiled, baked, and fried potatoes.

**FIGURE 1:**
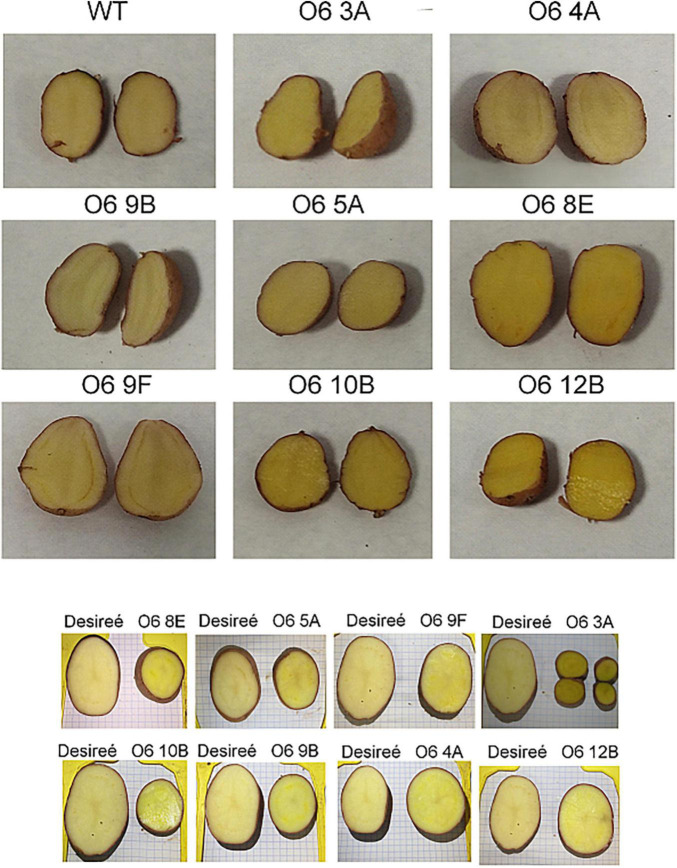
Potato wild type (Cv desirée) and transgenic lines accumulating the characteristic apocarotenoids in saffron.

For this purpose and to avoid possible pleiotropic effects on plant development derived by the use of a constitutive promoter, the construct was engineered to contain the tuber-specific patatin promoter and the minimum of genes needed to obtain the main saffron apocarotenoids, crocins, and picrocrocin. In this way, a binary construct was assembled with the Goldenbraid strategy (59, 60), carrying the *CsCCD2L*, *UGT74AD1*, and *UGT709G1* coding sequences driven by the patatin promoter, together with the hygromycin gene as a selection marker (Supplementary Figure 2). The transgenes were then introduced into *S. tuberosum* cv. Désirée via *A. tumefaciens*-mediated transformation. Approximately 20 independents transgenic lines of *S. tuberosum* cv. Désirée were generated, and the genomic DNA was further verified using PCR to check for the presence of the *CsCCD2L* transgene cassettes. In addition, eight lines were chosen for detailed analysis based on visual assessment of tuber color (Figure 1).

### Carotenoid content in transgenic tubers

Typically, white-fleshed potato tubers have low quantities of carotenoids, mainly β–β– and ε–β–xanthophylls (67). In order to evaluate alterations in apocarotenoid precursors, the selected transgenic tubers were screened for the content of carotenoids in tubers by HPLC-APCI-HRMS. In the WT control tubers, the carotenoids identified were antheraxanthin, zeaxanthin, violaxanthin, neoxanthin, and lutein in that order of decreasing amounts, while in transgenic lines generated with the O6 construct, none of these compounds were detected (Table 1), suggesting that somehow the presence of *CsCCD2L* depletes the existing low carotenoid pool for the production of crocetin, picrocrocin, and crocins. Interestingly, the levels of carotenoids generated in the initial steps of the metabolic pathway such as phytoene and phytofluene accumulate in lines O6 4A, O6 8E, and O6 9B (Table 1).

**TABLE 1:** Carotenoids identified and quantified in tubers of wild type (WT) and O6 transgenic lines.

Carotenoid	WT AVG ± SD	O6-3A AVG ± SD	O6-4A AVG ± SD	O6-5C AVG ± SD	O6-8E AVG ± SD	O6-9B AVG ± SD	O6-9F AVG ± SD	O6-10B AVG ± SD	O6-12C AVG ± SD
Antheraxanthin	0.091 ± 0.002	–	–	–	–	–	–	–	–
Zeaxanthin	0.069 ± 0.015	–	–	–	–	–	–	–	–
Violaxanthin	0.053 ± 0.002								
Neoxanthin	0.053 ± 0.005								
Lutein	0.004 ± 0.0003	–	–	–	–	–	–	–	–
Phytoene	–	–	0.033 ± 0.007	0.004 ± 0.001	0.017 ± 0.003	0.006 ± 0.003	–	–	–
Phytofluene	–	–	0.011 ± 0.002	–	0.012 ± 0.002	0.003 ± 0.002	–	–	–

Data are expressed as Fold Internal Standard (IS).

### Crocin and picrocrocin accumulation in transgenic tubers

**FIGURE 2:**
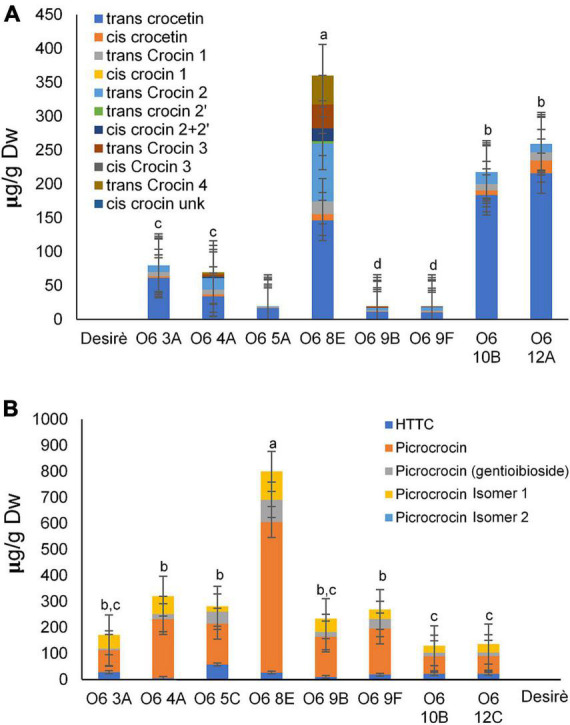
Metabolic engineering of saffron apocarotenoid in potato tubers. **(A)** Crocetin, crocin, and picrocrocin contents in the wild type (WT) and transgenic lines. **(B)** HTCC and picrocrocin derivative contents in the WT and transgenic lines. Different letters represent statistically significant differences of means, of transgenic lines, according to factorial analysis of variance (ANOVA) (*P* ≤ 0.01) (Tukey test). Error bars represent SD.

Polar extracts from the eight lines expressing the O6 construct were selected and analyzed for the *de novo* production of the main saffron apocarotenoids. Crocin identification and quantification were carried out by HPLC-DAD using the method described by Diretto et al. (34). In general, all lines contained all the apocarotenoids found in saffron, albeit at variable levels. All transgenic tuber potato lines accumulated high levels of crocins, including crocetin, crocin 1 [(crocetin-(β-D-glucosyl)-ester)] and crocin 2 [(crocetin)-(β-D-glucosyl)-(β-D-glucosyl)-ester)] (Figure 2A). In addition, lines from O6 4A, O6 8E, O6 9B, and O6 9F also revealed the presence of cis crocin 2 [(crocetin-(β-D-glucosyl)-(β-D-glucosyl)-ester)] and trans-crocin 4 [(crocetin-(β-D-gentiobiosyl)-(β-D-gentiobiosyl)-ester)]. Furthermore, trans-crocin 3 [(crocetin-(β-D-gentiobiosyl)-(β-D-glucosyl)-ester)] was found in lines O6 4A, O6 8E, and O6 9B.

The levels of picrocrocin, and its precursor HTCC, were also analyzed in WT and O6 transgenic tubers (Figure 2B). HTCC and picrocrocin were found in all of the tested lines but were not found in the WT tuber. Safranal together with other apocarotenoid volatiles were analyzed by GC-MS. In general, five different volatiles derived from carotenoids were detected, and their relative abundance is shown in Table 2. Notably, safranal, the metabolite produced from the 7,8:7’,8’ cleavage activity over zeaxanthin, was found to be a novel compound in the tuber potato carrying the O6 construct and was detected in all the transgenic lines. Additional volatile apocarotenoids derived from the activity of CCD1 and CCD4 such as β-damascenone, geranylacetone, and β-ionone showed a similar pattern of accumulation in the different transgenic lines. Overall, line O6 8E was the elite line and exhibited the highest levels of crocins and picrocrocin and its precursor HTCC, reaching 360 and 800 μg/g DW, respectively.

**TABLE 2:** Volatiles emitted by tuber potatoes of wild type (WT) and O6 transgenic lines.

Compounds	O6 3A	O6 4A	O6 5C	O6 8E	O6 9B	O6 9F	O6 10B	O6 12C	WT
6-Methyl-5-hepten-2-one	1.26 ± 0.74	0.96 ± 0.33	0.85 ± 0.51	0.72 ± 0.39	0.94 ± 0.47	1.03 ± 0.28	0.92 ± 0.29	1.09 ± 0.06	1.00 ± 0.46
Safranal*	1.67 ± 0.54	1.28 ± 0.49	2.00 ± 0.87	1.24 ± 0.21	1.00 ± 0.05	1.18 ± 0.15	1.72 ± 0.52	1.93 ± 0.32	NA
β-damascenone	1.06 ± 0.67	0.55 ± 0.68	0.73 ± 0.41	0.69 ± 0.39	0.35 ± 0.50	1.12 ± 0.58	0.79 ± 0.40	0.63 ± 0.46	1.00 ± 0.13
Geranylacetone	1.93 ± 0.40	3.66 ± 1.44	1.70 ± 0.83	1.07 ± 0.57	1.71 ± 0.70	1.10 ± 0.11	1.18 ± 0.40	1.49 ± 0.69	1.00 ± 0.40
β-ionone	0.37 ± 0.11	0.77 ± 0.44	0.20 ± 0.48	0.20 ± 0.35	0.87 ± 0.24	0.39 ± 0.56	0.67 ± 0.23	1.02 ± 0.26	1.00 ± 0.11

Results are presented as the ratio of transgenic lines vs. WT. Three plants per line were analyzed. Standard error (SD) represents three determinations. NA, not applicable. *This compound was undetectable (NA) in WT potato and the ratio was calculated as transgenic lines vs. line O6 8B, with this line showing the lowest levels of this metabolite among all the lines analyzed.

**FIGURE 3:**
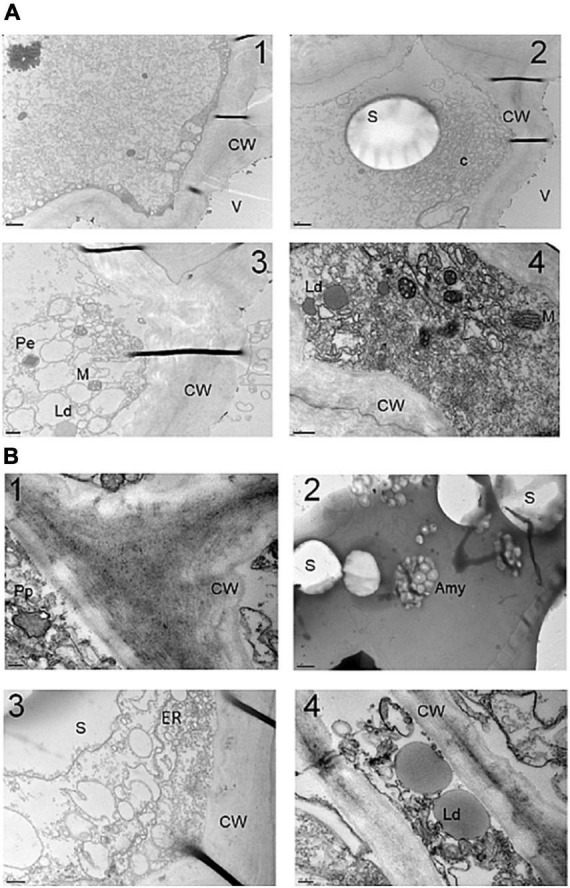
Transmission electron microscopy (TEM) of storage parenchyma cells of potato tubers. **(A)** TEM images of wild type (WT) simples. **(B)** TEM images of transgenic O6 line. Amy, amyloplast; C, clusters of amyloplast protusions; Cw, cell wall; Ld, lipid droplet; M, mitochondria; Pp, proplastid; Pe, peroxisome; ER, endoplasmic reticulum; V, vacuole; S, starch. Scale bars in panels (B1,4) are 200 μm, in panel (B2) is 2 μm, in panels (A1,2) are 1 μm, and in panels (A3,4,B3) are 0.5 μm.

In order to determine whether the accumulation of crocins was accompanied by changes at the structural level, the storage parenchyma cells of potato tubers of line O6 8E were analyzed. Large amyloplasts containing starch grains are prominent features of these potato tuber cells (68). An overview of the structure of these cells is presented in Figure 3, where different cell compartments are visualized, as mitochondria, peroxisomes, vacuoles, and endoplasmic reticulum (Figures 3A,B). The cells of potato tubers were highly vacuolated and possessed numerous amyloplasts containing starch granules (Figures 3A1,2,B2). More in detail, in WT control potato tuber cells, the amyloplasts primarily comprised one starch grain per plastid (Figure 3A2), whereas, in the O6 transgenic tissues, plastids were visualized with many small starch grains (Figure 3B2). Finally, lipid droplets of different sizes were also observed in WT samples (Figure 3A4) and in the transgenic tubers (Figure 3B4).

### Expression of genes associated with carotenoid metabolism

**FIGURE 4:**
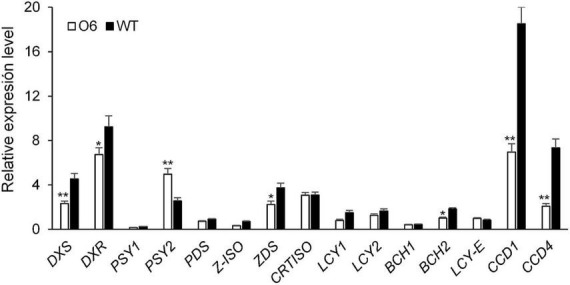
Expression of endogenous carotenoid and apocarotenoid genes in transgenic and wild type (WT) potato tubers. DXS, 1-deoxy-D-xylulose-5-phosphate synthase; DXR, 1-deoxy-D-xylulose-5-phosphate reductase; PSY1, phytoene synthase 1; PSY2, phytoene synthase 2; PDS, phytoene desaturase; LCY-b, lycopene β-cyclase; LCY-e, lycopene ∈-cyclase; BCH, β-carotene hydroxylase; CCD1, carotenoid cleavage dioxygenase 1; CCD4, carotenoid cleavage dioxygenase 4. qRT-PCR was performed in triplicate with three biological repeats. Error bars represent SD. *,**Significant differences from WT at *P* < 0.05 and *P* < 0.01, respectively, by Student’s *t*-test.

To determine whether the expression of the O6 construct affects the transcript levels of the endogenous carotenogenic genes in the transgenic tubers, the O6 8E line, characterized by the highest crocin and picrocrocin accumulation, was selected and a qRT-PCR was carried out to analyse a panel of 15 genes associated with carotenoid metabolism. Overall, expression of the transgene did influence expression of carotenoid metabolic genes (Figure 4). Indeed, and although expression of *DXS*, *DXR*, *PSY1*, *PSY2*, *PDS*, *ZDS*, *Z-ISO*, *LYC-b*, *BCH1*, and *BCH2* was slightly decreased, the most striking changes regarded the transcripts of *CCD1* and *CCD4*, which were clearly down-regulated (Figure 4). Interestingly, there was an inverse relationship between *CCD4* and *CCD1* expression and tuber carotenoid content in mature tubers. This down-regulation, particularly of the potato *CCD4* gene, is thought to increase carotenoid levels by lowering carotenoid enzymatic breakdown (58). Notably, the only gene whose expression was upregulated in transgenic tubers was *PSY2*, with results which increased by two-folds (Figure 4).

### Total antioxidant activity determination by DPPH assay

**FIGURE 5:**
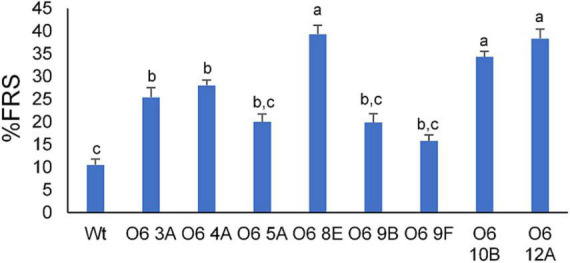
DPPH assay from transgenic and wild type (WT) potato tubers. Different letters represent statistically significant differences of means, according to factorial analysis of variance (ANOVA) (*P* ≤ 0.01) (Tukey test). Data represent the mean of three independent experiments ± SD.

The DPPH method is often used for the assessment of free radical-scavenging capacities due to its simplicity, stability, accuracy, and reproducibility. Interestingly, all transgenic tubers had strong antioxidant activity as measured by their capacity to scavenge free radicals (Figure 5). Compared with the tuber of WT, indeed, the antioxidant capacity of these transgenic line tubers was around 2 to 7-fold greater. In general, lines with a high content in crocins showed higher antioxidant activity. Line O6 8E extracts exhibited lower IC50 values, thus indicating higher radical scavenging activity in comparison with the other lines, while line O6 9F displayed the lowest antioxidant activity.

### Cooking affects the content of crocins and crocetin in the potato samples

**FIGURE 6:**
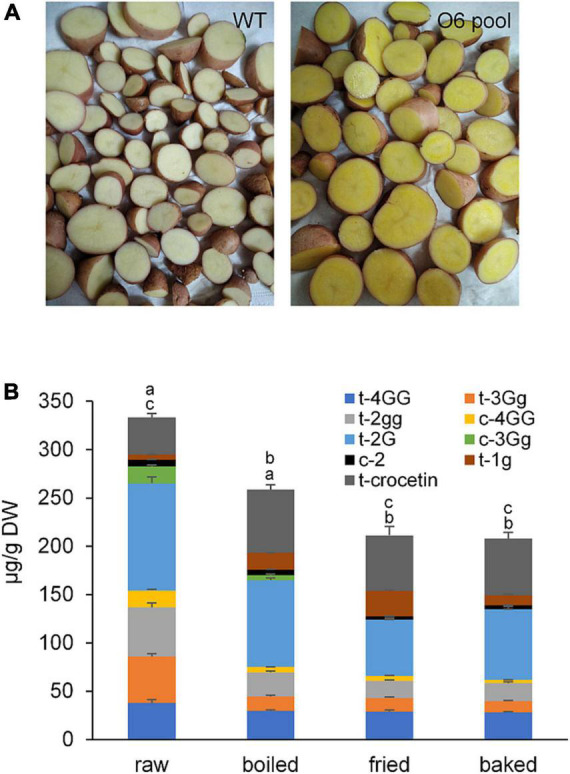
Potatoes (cv. Desirée) used for cooked stability of crocins and bioaccessibility experiments. **(A)** Internal phenotype of wild type (WT) (cv. Desirée) potato tubers and O6 transgenic (cv. Desirée) potato tuber pool. **(B)** Crocetin and crocetin ester contents in raw and cooked potato tubers accumulating the characteristic apocarotenoids in saffron. Different letters represent statistically significant differences of means, of cooking procedures, according to factorial analysis of variance (ANOVA) (*P* ≤ 0.05) (Tukey test). Above letters in correspond to differences in total crocins content, and letters below correspond to differences in crocetin content.

The contents of crocins and crocetin in raw potatoes and in the three cooked products were evaluated and shown in Figure 6. Overall, the highest content of total crocins was found in raw potatoes (249.00 ± 20.13 μg/g DW) and decreased with the cooking method from 193.99 ± 11.21 μg/g dry matter in boiled to 149.43 ± 12.94 μg/g DW in baked potatoes (Figure 6). However, the crocetin content followed an inverse result from 58.55 ± 6.31 μg/g dry matter in baked to 65.36 ± 5.25 μg/g DW in boiled, with the lowest content present in raw potatoes (38.44 ± 3.86 μg/g DW). The increase in crocetin might be due to the hydrolysis of sugars from the crocin molecules due to the high temperatures. Further, crocins and crocetin are sensitive to thermal treatment and to acidic environments (69). The t-3-Gg crocetin ester was the one suffering the major decrease in all the cooking treatments (Figure 6), with baking showing a reduction of more than 82% of the initial content in the raw potatoes. Thermal treatment was shown to affect crocins in different ways, making some of them more labile than others. This was the case of t-3-Gg, which is less stable than t-4-GG (70, 71). Under the cooking conditions used in this study, boiling appeared to be the best way to preserve crocins (Figure 6), with a loss of 17.44% in total apocarotenoids, while baking was the most destructive method with a loss of 44.81%. Overall, the findings revealed that different cooking procedures had a substantial effect on the preservation of apocarotenoids in potato tubers.

### Apocarotenoid bioaccessibility changes in potatoes submitted to different cooking methods

Previous studies have analyzed the bioaccessibility of saffron and gardenia methanolic extracts (72) or aqueous extracts (73), showing the impact of temperature and the acidic conditions on the stability of crocetin esters and in the degree of their isomerization. The analyses of the different fractions during the *in vitro* digestion by HPLC-DAD showed the presence of multiple isomers, as previously observed during the *in vitro* digestion of saffron extracts (72), where isomerization and degradation occur concurrently during the digestive process. Similar findings have been found for a variety of carotenoids, including lycopene (74), zeaxanthin, and lutein (75) as well as for norbixin, the apocarotenoid of *Bixa orellana*, reaching 90% of isomerization (76). Since the new isomers could not be individually quantified for comparison among the different cooking methods, the data were normalized by expressing the total ester crocetin content as the measurement of the extracts at 440 nm. Data reported in Table 3 shows the percentage of apocarotenoids (crocetin plus crocins) transferred from the potato samples into the medium after *in vitro* digestion (i.e., the bioaccessibility). In general, bioaccessibility of apocarotenoids was higher in boiled potatoes (97.23%) compared to raw, baked, and fried ones (80.97, 78.96, and 76.18% respectively). In general, crocetin ester bioaccessibility is found to be high when compared to that reported for hydrophobic carotenoids (77), as also is the case of β-carotene and xanthophyll-enriched potato tubers (78). In this context, we can rule out the possibility that crocetin esters hydrolyze into crocetin after reaching the small intestine and are absorbed into blood circulation as previously observed (79, 80).

**TABLE 3:** Apocarotenoid bioaccessibility (%) in different cooking treatments.

Types of cooking g methods	Gastric digestion	Intestinal digestion	Non-digested residue
Raw	13.01 ± 0.76	67.97 ± 5.82	11.01 ± 2.31
boiling	21.66 ± 5.12	75.57 ± 4.32	6.71 ± 0.41
frying	16.57 ± 3.87	59.61 ± 6.30	7.24 ± 1.27
baking	17.16 ± 0.53	61.80 ± 3.82	14.41 ± 2.46

Data are means of three independent determinations.

## Discussion

Plant apocarotenoids have a huge beneficial impact on human health, by preventing or managing chronic disease or its symptoms. In addition, apocarotenoids have received considerable attention because of their biotechnological applications in the fields of food processing, pharmaceuticals, cosmetics, perfume, and agriculture (81). In this context, crocins, picrocrocin, and safranal show bioactive properties with several therapeutical and pharmacological applications (66). On the other hand, potato is the third-largest food crop in the world, and its nutritional value is quite essential, especially, regarding the importance of carotenoids and apocarotenoids in the diet (82). Unfortunately, potato germplasm does not contain a good source in β-carotene, zeaxanthin, or other xanthophylls (83). Notably, while several high carotenoid potatoes have been achieved, no studies up to now have reported successful production of high value apocarotenoid-enriched tubers. In this context, the primary goal of this study was to generate tuber potatoes accumulating the bioactive metabolites: crocins, picrocrocin, and safranal. By using a combinatorial strategy in which *CsCCD2L* was introduced with *UGT74AD1* and *UGT709G1* driven by the patatin: promoter, it was possible to accumulate crocins, picrocrocin, and safranal in the potato tuber (Figure 1). Previously, different platforms have been used to generate these valuable metabolites by expressing *CsCCD2L* in different plant systems. Transient expression of this gene in *N. benthamiana* leaves, allowed for obtaining up to 30.5 μg/g DW of crocins and 4.13 μg/g DW of picrocrocin (34). A transitory system using virus-driven expression of *CsCCD2L* enabled the accumulation of 2.18 ± 0.23 mg/g DW of crocins and 8.24 ± 2.93 mg/g DW of picrocrocin (41). More recently, *CsCCD2L* was stably expressed into *N. tabacum*, *N. glauca* and tomato. In *N. glauca* crocins reached almost 400 μg/g DW in leaves, while in *N. tabacum* it was 36 μg/g DW (42). On the contrary, and notably, in ripe tomatoes, levels of crocins up to 14.48 mg/g DW were achieved (43). Our transgenic potato tubers showed an accumulation of crocins and picrocrocin of 360 and 800 μg/g DW, respectively. The fluctuation in the amount of these bioactive metabolites could be attributed mainly to the concentrations of carotenoids present in these species, a reduced pool of carotenoids in tubers of cv. Desirée (around 5.90 ± 1.47 μg/g DW) compared to those present in tomato plants (around 2.85 mg/g DW). Moreover, it must be remarked that tomato fruit has well established sub-cellular structures (chromoplasts) to accumulate and sequestrate high carotenoid precursor amounts, with a good plasticity capacity to remodel their metabolite profiles upon genetic engineering attempt. On the contrary, potato tubers grown in the dark only contain amyloplasts, which can be converted into chloroplast but not into chromoplasts when moved to light conditions (84), thus suggesting a higher constraint extent in terms of carotenoid synthesis modulation. In addition, the type of carotenoids present in these tissues and their contribution to the pool of precursors for the biosynthesis of saffron apocarotenoids would also be important. Whereas in tomato, indeed, the major carotenoids found are present in the pathway previous to zeaxanthin; in potato the major carotenoid types are violaxanthin and lutein. Analysis of our data in relation to the synthesis of other potentially useful compounds in potato tubers, revealed the successful outcome of this apocarotenoid metabolic engineering: indeed, potato lines accumulating ketocarotenoid expressing the algal *bkt1* gene, encoding a β-ketolase, showed an amount of 14 μg/g DW (85), whereas developing and mature tubers of transgenic *crtB* and *crtB* + *crtI* + *crtY* Desirée lines reached, respectively, c. 11 and 47 μg/g DW of β-carotene (53, 55, 86); while tubers overexpressing the *Orange* (*Or*) gene, involved in chromoplast differentiation accumulate (87) up to 30 μg/g DW of β-carotene after 5 months storage in cold rooms; and, more recently, transgenic lines with stable expression of *CrtRb2* and *PSY2* boosted the total carotenoid to around 25 μg/g DW (88).

The carotenoids detected in WT control tubers were antheraxanthin, zeaxanthin, violaxanthin, neoxanthin, and lutein in that order of amount, while in the transgenic tubers none of these carotenoids were identified. It is known that CsCCD2L recognized zeaxanthin and lutein as substrates (26, 27), and its activity in the O6 transgenic tubers could explain the absence of these carotenoids, which were cleaved and, for the former, used in the production of saffron apocarotenoids. By contrast, phytoene and phytofluene levels were enhanced in the transgenic tubers, which might result from the increased expression of *PSY2* in the transgenic lines. PSY is thought to be a rate-limiting enzyme in the biosynthesis of carotenoids, and altering its expression or activity changes flux through the pathway. In fact, previous studies revealed that PSY2 is a key enzyme in carotenoid biosynthesis in potato, as revealed by both genetic and transgenic studies (53, 89). In this context it was not surprising to find high levels of *PSY2* transcripts in transgenic potato tubers. Notably, an increase in phytoene and phytofluene has also been observed in *N. benthamiana* leaves transformed with *CsCCD2* (41), while a strong reduction of these early carotenoids in apocarotenoid-enriched ripe tomatoes has been recorded (43). This distinct attitude might evidence a different capacity in terms of carotenoid regulation in vegetative- and reproductive-type organs, and/or, as previously mentioned, could be associated to the divergent carotenoid metabolism occurring in the different organelles (amyloplasts/chloroplasts vs. chromoplasts). As opposed to the increased expression of *PSY2*, the transcript amounts of two genes related to carotenoid catabolism were decreased. More in detail, *CCD1* and *CCD4* expression levels were reduced in the transgenic tubers. CCD1 enzymes have been proposed to act as scavengers of carotenoids that have been depleted by non-enzymatic oxidation (90) and their transcript level in potato tubers has been found to be inversely correlated with carotenoid accumulation (91). Thus, their reduced expression in our transgenic tubers might entail an increase in the carotenoid pool for the biosynthesis of saffron apocarotenoids. Additionally, it has been demonstrated that down-regulating the potato *CCD4* gene can raise the carotenoid concentration (58). This is in agreement with its enzymatic characteristics: more specifically, the substrates of CCD4 in potatoes are β-carotene, α-carotene, and lutein (92). The fall in *CCD4* transcripts could suggested that the activity of CsCCD2L is able to intercept the metabolic flux toward the production of crocins and picrocrocin, from these upstream metabolites.

Some authors point out the antioxidant activity indistinctly to any of the components of saffron, while others attribute these benefits to the total aqueous extract of saffron and crocins (93, 94). Besides, similarity in the structure of metabolite comparison between crocins and crocetin revealed that crocetin showed a higher antioxidant efficacy than crocins. This might be caused by the gentobiose sugar presence in the molecular structure of crocins, which alters the degree of conjugation of the active compound and subsequently may vary its antioxidant activity (95). The presence of saffron apocarotenoids in the transgenic tubers led to an increase in the antioxidant activity of cv. Desirée tubers. Previous studies have shown that anthocyanins present in the peel of cv. Desirée tubers mainly contribute to their antioxidant properties, although the peel is largely discarded during potato consumption and processing (96). On the other hand, the amount of carotenoid in the flesh is clearly related to its antioxidant capacity, with values up to 20-fold higher in deeply yellow to orange-fleshed cultivars (97). Thus, the fortified potatoes with saffron apocarotenoids resulted in boosted antioxidant activity of varieties with an intrinsic low carotenoid content.

Usually, foods that have been heated have considerable modifications in their bioactive compounds. Depending on the method used and the nature of the metabolite, distinct patterns were seen when the transgenic tubers were cooked applying different techniques. The highest amount of total crocins detected was in raw, boiled, fried, and baked tubers in that order, while the ratio between crocetin and crocins was inversely proportional to the high concentration of total crocins. Data revealed that all crocins examined were unstable under the various conditions of cooking, especially trans-crocin 3. Several reports have demonstrated the degradation and the loss of crocins during the exposition to higher temperatures (71, 98, 99). Under the cooking conditions used in this study, boiling seemed to be the best way to keep crocins intact. Only 17.44% in total apocarotenoids was missing after boiling, while baking was the worst treatment: inducing a loss of 44.81%. Heat significantly reduced the content of crocins from dry stigmas by over 50% after 30 mins of heating (71). However, 83% of the total crocins from tuber potatoes remained unharmed after the boiling method, which could be a consequence of the nature of potato tissue where the crocins are more protected.

Many scientific studies have analyzed the bioaccesibility of saffron and gardenia methanolic extracts or aqueous extracts, showing the impact of temperature and acidic conditions on the stability of crocetin esters and in the degree of isomerization. As stated previously, crocins and crocetin are unstable and show low bioavailability (95). After oral administration, crocins are transformed into crocetin in the intestinal tract by the action of the enzymes of the intestinal epithelium (100). Using saffron extracts, only 50% of crocins were reported to be bioaccessible under *in vitro* digestion conditions while the concentration of crocetin was 10 times higher than the crocins detected (72, 101). Overall, our results revealed that the bioaccessibility of apocarotenoids using boiled potatoes was higher compared to raw, baked, and fried ones, reaching 97.23%, which is also higher than the carotenoid retention and bioaccessibility reported for β-carotene in *Or*-overexpressing lines (15%) (87), and β-carotene and total carotenoid-enriched “golden” tubers, equal to, respectively, 80 and 20% (78). Thus, it can be speculated that boiling potatoes is the best way to preserve crocins almost intact and seems to be a more appropriate matrix than an aqueous extract of saffron in terms of bioaccessibility.

## Data availability statement

The original contributions presented in this study are included in this article/Supplementary material, further inquiries can be directed to the corresponding authors.

## Author contributions

LG, AG, and OA conceived and designed the research and drafted the manuscript. AL-J, LM, EN, ÁR-M, JR, GD, SF, JA, AG, SP, RL, AM-B, LG, and OA conducted the experiments. LG, AG, GD, and OA analyzed the data. All authors reviewed and contributed to the final manuscript which was approved by all.

## Footnotes

1
http://pubchem.ncbi.nlm.nih.gov/

2
http://fiehnlab.ucdavis.edu/staff/kind/Metabolomics/MS-Adduct-Calculator/

## References

[1] Britton G. Overview of carotenoid biosynthesis. *Carotenoids.* (1998) 3:13–147.

[2] Cunningham F Jr Gantt E. Genes and enzymes of carotenoid biosynthesis in plants. *Annu Rev Plant Biol.* (1998) 49:557–83. 10.1146/annurev.arplant.49.1.557 15012246

[3] DellaPenna D Pogson BJ. Vitamin synthesis in plants: tocopherols and carotenoids. *Annu Rev Plant Biol.* (2006) 57:711–38. 10.1146/annurev.arplant.56.032604.144301 16669779

[4] Fraser PD Bramley PM. The biosynthesis and nutritional uses of carotenoids. *Prog Lipid Res.* (2004) 43:228–65. 10.1016/j.plipres.2003.10.002 15003396

[5] Hirschberg J. Carotenoid biosynthesis in flowering plants. *Curr Opin Plant Biol.* (2001) 4:210–8. 10.1016/S1369-5266(00)00163-1 11312131

[6] Armstrong GA. Genetics of eubacterial carotenoid biosynthesis: a colorful tale. *Annu Rev Microbiol.* (1997) 51:629–59. 10.1146/annurev.micro.51.1.629 9343362

[7] Avalos J Cerdá-Olmedo E. Fungal carotenoid production. *Handb Fungal Biotechnol.* (2004) 20:367–78. 10.1201/9780203027356.ch28

[8] de Carvalho CC Caramujo MJ. Carotenoids in aquatic ecosystems and aquaculture: a colorful business with implications for human health. *Front Mar Sci.* (2017) 4:93. 10.3389/fmars.2017.00093

[9] Weaver RJ Santos ES Tucker AM Wilson AE Hill GE. Carotenoid metabolism strengthens the link between feather coloration and individual quality. *Nat Commun.* (2018) 9:1–9. 10.1038/s41467-017-02649-z 29311592 PMC5758789

[10] Maoka T. Carotenoids as natural functional pigments. *J Nat Med.* (2020) 74:1–16. 10.1007/s11418-019-01364-x 31588965 PMC6949322

[11] Ahrazem O Gomez-Gomez L Rodrigo MJ Avalos J Limon MC. Carotenoid cleavage oxygenases from microbes and photosynthetic organisms: features and functions. *Int J Mol Sci.* (2016) 17:1781. 10.3390/ijms17111781 27792173 PMC5133782

[12] Rodríguez A Alquézar B Peña L. Fruit aromas in mature fleshy fruits as signals of readiness for predation and seed dispersal. *New Phytol.* (2013) 197:36–48. 10.1111/j.1469-8137.2012.04382.x 23127167

[13] Macias FA Lopez A Varela RM Torres A Molinillo JM. Bioactive Apocarotenoids annuionones F and G: structural revision of annuionones a, B and E. *Phytochemistry.* (2004) 65:3057–63. 10.1016/j.phytochem.2004.08.048 15504441

[14] DellaGreca M Di Marino C Zarrelli A D’Abrosca B. Isolation and phytotoxicity of apocarotenoids from chenopodium album. *J Nat Prod.* (2004) 67:1492–5. 10.1021/np049857q 15387648

[15] Vishwakarma K Upadhyay N Kumar N Yadav G Singh J Mishra RK Abscisic acid signaling and abiotic stress tolerance in plants: a review on current knowledge and future prospects. *Front Plant Sci.* (2017) 8:161. 10.3389/fpls.2017.00161 28265276 PMC5316533

[16] Kapulnik Y Delaux PM Resnick N Mayzlish-Gati E Wininger S Bhattacharya C Strigolactones affect lateral root formation and root-hair elongation in arabidopsis. *Planta.* (2011) 233:209–16. 10.1007/s00425-010-1310-y 21080198

[17] Harrison EH Quadro L. Apocarotenoids: emerging roles in mammals. *Annu Rev Nutr.* (2018) 38:153–72. 10.1146/annurev-nutr-082117-051841 29751734 PMC6295211

[18] Meléndez-Martínez AJ. An overview of carotenoids, apocarotenoids, and vitamin a in agro-food, nutrition, health, and disease. *Mol Nutr Food Res.* (2019) 63:1801045. 10.1002/mnfr.201801045 31189216

[19] Rubio-Moraga A Castillo-Lopez R Gomez-Gomez L Ahrazem O. Saffron is a monomorphic species as revealed by Rapd, Issr and microsatellite analyses. *BMC Res Notes.* (2009) 2:189. 10.1186/1756-0500-2-189 19772674 PMC2758891

[20] Ahrazem O Rubio-Moraga A Nebauer SG Molina RV Gomez-Gomez L. Saffron: its phytochemistry, developmental processes, and biotechnological prospects. *J Agric Food Chem.* (2015) 63:8751–64. 10.1021/acs.jafc.5b03194 26414550

[21] Kanakis CD Daferera DJ Tarantilis PA Polissiou MG. Qualitative determination of volatile compounds and quantitative evaluation of safranal and 4-hydroxy-2,6,6-trimethyl-1-cyclohexene-1-carboxaldehyde (Htcc) in Greek saffron. *J Agric Food Chem.* (2004) 52:4515–21. 10.1021/jf049808j 15237960

[22] Ahrazem O Trapero A Gomez MD Rubio-Moraga A Gomez-Gomez L. Genomic analysis and gene structure of the plant carotenoid dioxygenase 4 family: a deeper study in *Crocus sativus* and its allies. *Genomics.* (2010) 96:239–50. 10.1016/j.ygeno.2010.07.003 20633636

[23] Castillo R Fernández JA Gómez-Gómez L. Implications of carotenoid biosynthetic genes in apocarotenoid formation during the stigma development of *Crocus sativus* and its closer relatives. *Plant Physiol.* (2005) 139:674–89. 10.1104/pp.105.067827 16183835 PMC1255987

[24] Rubio A Rambla JL Santaella M Gomez MD Orzaez D Granell A Cytosolic and plastoglobule-targeted carotenoid dioxygenases from *Crocus sativus* are both involved in beta-ionone release. *J Biol Chem.* (2008) 283:24816–25. 10.1074/jbc.M804000200 18611853 PMC3259819

[25] Ahrazem O Argandona J Fiore A Aguado C Lujan R Rubio-Moraga A Transcriptome analysis in tissue sectors with contrasting crocins accumulation provides novel insights into apocarotenoid biosynthesis and regulation during chromoplast biogenesis. *Sci Rep.* (2018) 8:2843. 10.1038/s41598-018-21225-z 29434251 PMC5809551

[26] Ahrazem O Rubio-Moraga A Berman J Capell T Christou P Zhu C The carotenoid cleavage dioxygenase Ccd2 catalysing the synthesis of crocetin in spring crocuses and saffron is a plastidial enzyme. *New Phytol.* (2016) 209:650–63. 10.1111/nph.13609 26377696

[27] Frusciante S Diretto G Bruno M Ferrante P Pietrella M Prado-Cabrero A Novel carotenoid cleavage dioxygenase catalyzes the first dedicated step in saffron crocin biosynthesis. *Proc Natl Acad Sci U.S.A.* (2014) 111:12246–51. 10.1073/pnas.1404629111 25097262 PMC4143034

[28] Ahrazem O Rubio-Moraga A Trapero A Gomez-Gomez L. Developmental and stress regulation of gene expression for a 9-Cis-epoxycarotenoid dioxygenase, Cstnced, isolated from *Crocus sativus* stigmas. *J Exp Bot.* (2012) 63:681–94. 10.1093/jxb/err293 22048040

[29] Rubio-Moraga A Ahrazem O Perez-Clemente RM Gomez-Cadenas A Yoneyama K Lopez-Raez JA Apical dominance in saffron and the involvement of the branching enzymes Ccd7 and Ccd8 in the control of bud sprouting. *BMC Plant Biol.* (2014) 14:171. 10.1186/1471-2229-14-171 24947472 PMC4077219

[30] Gómez-Gómez L Pacios LF Diaz-Perales A Garrido-Arandia M Argandoña J Rubio-Moraga Á Expression and interaction analysis among saffron aldhs and crocetin dialdehyde. *Int J Mol Sci.* (2018) 19:1409. 10.3390/ijms19051409 29747375 PMC5983644

[31] Moraga AR Nohales PF Perez JA Gomez-Gomez L. Glucosylation of the saffron apocarotenoid crocetin by a glucosyltransferase isolated from *Crocus sativus* stigmas. *Planta.* (2004) 219:955–66. 10.1007/s00425-004-1299-1 15605174

[32] Gómez-Gómez L Parra-Vega V Rivas-Sendra A Seguí-Simarro JM Molina RV Pallotti C Unraveling massive crocins transport and accumulation through proteome and microscopy tools during the development of saffron stigma. *Int J Mol Sci.* (2017) 18:76. 10.3390/ijms18010076 28045431 PMC5297711

[33] Diretto G López-Jiménez AJ Ahrazem O Frusciante S Song J Rubio-Moraga Á Identification and characterization of apocarotenoid modifiers and carotenogenic enzymes for biosynthesis of crocins in *Buddleja davidii* flowers. *J Exp Bot.* (2021) 72:3200–18. 10.1093/jxb/erab053 33544822

[34] Diretto G Ahrazem O Rubio-Moraga A Fiore A Sevi F Argandona J Ugt709g1: a novel uridine diphosphate glycosyltransferase involved in the biosynthesis of picrocrocin, the precursor of safranal in saffron (*Crocus sativus*). *New Phytol.* (2019) 224:725–40. 10.1111/nph.16079 31356694

[35] Ahrazem O Diretto G Argandoña J Rubio-Moraga Á Julve JM Orzáez D Evolutionarily distinct carotenoid cleavage dioxygenases are responsible for crocetin production in *Buddleja davidii*. *J Exp Bot.* (2017) 68:4663–77. 10.1093/jxb/erx277 28981773

[36] Xu Z Pu X Gao R Demurtas OC Fleck SJ Richter M Tandem gene duplications drive divergent evolution of caffeine and crocin biosynthetic pathways in plants. *BMC Biol.* (2020) 18:63. 10.1186/s12915-020-00795-3 32552824 PMC7302004

[37] Ghadrdoost B Vafaei AA Rashidy-Pour A Hajisoltani R Bandegi AR Motamedi F Protective effects of saffron extract and its active constituent crocin against oxidative stress and spatial learning and memory deficits induced by chronic stress in rats. *Eur J Pharmacol.* (2011) 667:222–9. 10.1016/j.ejphar.2011.05.012 21616066

[38] Gohari AR Saeidnia S Mahmoodabadi MK. An overview on saffron, phytochemicals, and medicinal properties. *Pharmacogn Rev.* (2013) 7:61–6. 10.4103/0973-7847.112850 23922458 PMC3731881

[39] Toner C. Consumer perspectives about antioxidants. *J Nutr.* (2004) 134:3192S–3S. 10.1093/jn/134.11.3192S 15514303

[40] Fiedor J Burda K. Potential role of carotenoids as antioxidants in human health and disease. *Nutrients.* (2014) 6:466–88. 10.3390/nu6020466 24473231 PMC3942711

[41] Martí M Diretto G Aragonés V Frusciante S Ahrazem O Gómez-Gómez L Efficient production of saffron crocins and picrocrocin in nicotiana benthamiana using a virus-driven system. *Metab Eng.* (2020) 61:238–50. 10.1016/j.ymben.2020.06.009 32629020

[42] Ahrazem O Zhu C Huang X Rubio Moraga A Capell T Christou P Metabolic engineering of crocin biosynthesis in *Nicotiana* species. *Front Plant Sci.* (2022) 13:861140. 10.3389/fpls.2022.861140 35350302 PMC8957871

[43] Ahrazem O Diretto G Rambla JL Rubio-Moraga A Lobato MA Frusciante S Engineering high levels of saffron apocarotenoids in tomato. *Horticult Res.* (2022) 9:uhac074. 10.1093/hr/uhac074 35669709 PMC9157650

[44] López AJ Frusciante S Niza E Ahrazem O Rubio-Moraga Á Diretto G A new glycosyltransferase enzyme from family 91, Ugt91p3, Is responsible for the final glucosylation step of crocins in saffron (*Crocus sativus* L.). *Int J Mol Sci.* (2021) 22:8815. 10.3390/ijms22168815 34445522 PMC8396231

[45] Demurtas OC de Brito Francisco R Diretto G Ferrante P Frusciante S Pietrella M Abcc transporters mediate the vacuolar accumulation of crocins in saffron stigmas. *Plant Cell.* (2019) 31:2789–804. 10.1105/tpc.19.00193 31548254 PMC6881118

[46] Bvenura C Witbooi H Kambizi L. Pigmented potatoes: a potential panacea for food and nutrition security and health? *Foods.* (2022) 11:175. 10.3390/foods11020175 35053906 PMC8774573

[47] Elizalde-Romero CA Montoya-Inzunza LA Contreras-Angulo LA Heredia JB Gutiérrez-Grijalva EP. Solanum fruits: phytochemicals, bioaccessibility and bioavailability, and their relationship with their health-promoting effects. *Front Nutr.* (2021) 8:790582. 10.3389/fnut.2021.790582 34938764 PMC8687741

[48] Aversano R Contaldi F Adelfi MG D’Amelia V Diretto G De Tommasi N Comparative metabolite and genome analysis of tuber-bearing potato species. *Phytochemistry.* (2017) 137:42–51. 10.1016/j.phytochem.2017.02.011 28215419

[49] Tai HH Worrall K Pelletier Y De Koeyer D Calhoun LA. Comparative metabolite profiling of *Solanum tuberosum* against six wild *Solanum* species with colorado potato beetle resistance. *J Agric Food Chem.* (2014) 62:9043–55. 10.1021/jf502508y 25144460

[50] Tatarowska B Milczarek D Wszelaczyńska E Pobereżny J Keutgen N Keutgen AJ Carotenoids variability of potato tubers in relation to genotype, growing location and year. *Am J Potato Res.* (2019) 96:493–504. 10.1007/s12230-019-09732-9

[51] Diretto G Tavazza R Welsch R Pizzichini D Mourgues F Papacchioli V Metabolic engineering of potato tuber carotenoids through tuber-specific silencing of lycopene epsilon cyclase. *BMC Plant Biol.* (2006) 6:13. 10.1186/1471-2229-6-13 16800876 PMC1570464

[52] Diretto G Welsch R Tavazza R Mourgues F Pizzichini D Beyer P Silencing of beta-carotene hydroxylase increases total carotenoid and beta-carotene levels in potato tubers. *BMC Plant Biol.* (2007) 7:11. 10.1186/1471-2229-7-11 17335571 PMC1828156

[53] Ducreux LJ Morris WL Hedley PE Shepherd T Davies HV Millam S Metabolic engineering of high carotenoid potato tubers containing enhanced levels of β-carotene and lutein. *J Exp Bot.* (2005) 56:81–9. 10.1093/jxb/eri016 15533882

[54] Lu S Van Eck J Zhou X Lopez AB O’Halloran DM Cosman KM The cauliflower or gene encodes a Dnaj cysteine-rich domain-containing protein that mediates high levels of β-carotene accumulation. *Plant Cell.* (2006) 18:3594–605. 10.1105/tpc.106.046417 17172359 PMC1785402

[55] Diretto G Al-Babili S Tavazza R Papacchioli V Beyer P Giuliano G. Metabolic engineering of potato carotenoid content through tuber-specific overexpression of a bacterial mini-pathway. *PLoS One.* (2007) 2:e350. 10.1371/journal.pone.0000350 17406674 PMC1831493

[56] Sulli M Mandolino G Sturaro M Onofri C Diretto G Parisi B Molecular and biochemical characterization of a potato collection with contrasting tuber carotenoid content. *PLoS One.* (2017) 12:e0184143. 10.1371/journal.pone.0184143 28898255 PMC5595298

[57] Milczarek D Tatarowska B. Evaluation of potato cultivars and breeding lines for carotenoids content in tubers. *Plant Breed Seed Sci.* (2017) 75:11–6. 10.1515/plass-2017-0003 31547486

[58] Campbell R Ducreux LJ Morris WL Morris JA Suttle JC Ramsay G The metabolic and developmental roles of carotenoid cleavage dioxygenase4 from potato. *Plant Physiol.* (2010) 154:656–64. 10.1104/pp.110.158733 20688977 PMC2949026

[59] Sarrion-Perdigones A Palaci J Granell A Orzaez D. Design and construction of multigenic constructs for plant biotechnology using the goldenbraid cloning strategy. *Methods Mol Biol.* (2014) 1116:133–51. 10.1007/978-1-62703-764-8_10 24395362

[60] Sarrion-Perdigones A Vazquez-Vilar M Palaci J Castelijns B Forment J Ziarsolo P Goldenbraid 2.0: a comprehensive DNA assembly framework for plant synthetic biology. *Plant Physiol.* (2013) 162:1618–31. 10.1104/pp.113.217661 23669743 PMC3707536

[61] Diretto G Rubio-Moraga A Argandona J Castillo P Gomez-Gomez L Ahrazem O. Tissue-specific accumulation of sulfur compounds and saponins in different parts of garlic cloves from purple and white ecotypes. *Molecules.* (2017) 22:1359. 10.3390/molecules22081359 28825644 PMC6152257

[62] Tavazza R Tavazza M Ordas RJ Ancora G Benvenuto E. Genetic transformation of potato (*Solanum tuberosum*): an efficient method to obtain transgenic plants. *Plant Sci.* (1989) 59:175–81. 10.1016/0168-9452(89)90135-0

[63] Dono G Rambla JL Frusciante S Fabene E Gómez-Cadenas A Granell A Pigment-related mutations greatly affect berry metabolome in san marzano tomatoes. *Horticulturae.* (2022) 8:120. 10.3390/horticulturae8020120

[64] Lopez AB Van Eck J Conlin BJ Paolillo DJ O’Neill J Li L. Effect of the cauliflower or transgene on carotenoid accumulation and chromoplast formation in transgenic potato tubers. *J Exp Bot.* (2008) 59:213–23. 10.1093/jxb/erm299 18256051

[65] Raines C Traynor M Ingram J. *Experimental Botany in 2017.* Oxford: Oxford University Press (2017). p. 347–9. 10.1093/jxb/erw504 PMC5444439 28201652

[66] Mykhailenko O Kovalyov V Goryacha O Ivanauskas L Georgiyants V. Biologically active compounds and pharmacological activities of species of the genus *Crocus*: a review. *Phytochemistry.* (2019) 162:56–89. 10.1016/j.phytochem.2019.02.004 30856530

[67] Fernandez-Orozco R Gallardo-Guerrero L Hornero-Méndez D. Carotenoid profiling in tubers of different potato (*Solanum* Sp) cultivars: accumulation of carotenoids mediated by xanthophyll esterification. *Food Chem.* (2013) 141:2864–72. 10.1016/j.foodchem.2013.05.016 23871035

[68] Borucki W Bederska M Sujkowska-Rybkowska M. Visualisation of plastid outgrowths in potato (*Solanum tuberosum* L.) tubers by carboxyfluorescein diacetate staining. *Plant Cell Rep.* (2015) 34:853–60. 10.1007/s00299-015-1748-2 25627254 PMC4405334

[69] Tsimidou M Tsatsaroni E. Stability of saffron pigments in aqueous extracts. *J Food Sci.* (1993) 58:1073–5. 10.1111/j.1365-2621.1993.tb06116.x

[70] Sánchez AM Carmona M Ordoudi SA Tsimidou MZ Alonso GL. Kinetics of individual crocetin ester degradation in aqueous extracts of saffron (*Crocus sativus* L.) upon thermal treatment in the dark. *J Agric Food Chem.* (2008) 56:1627–37. 10.1021/jf0730993 18237133

[71] Rodríguez-Neira L Lage-Yusty MA López-Hernández J. Influence of culinary processing time on Saffron’s bioactive compounds (*Crocus sativus* L.). *Plant Foods Hum Nutr.* (2014) 69:291–6. 10.1007/s11130-014-0447-4 25373843

[72] Kyriakoudi A Tsimidou MZ O’Callaghan YC Galvin K O’Brien NM. Changes in total and individual crocetin esters upon in vitro gastrointestinal digestion of saffron aqueous extracts. *J Agric Food Chem.* (2013) 61:5318–27. 10.1021/jf400540y 23654200

[73] Ordoudi SA Kyriakoudi A Tsimidou MZ. Enhanced bioaccessibility of crocetin sugar esters from saffron in infusions rich in natural phenolic antioxidants. *Molecules.* (2015) 20:17760–74. 10.3390/molecules201017760 26404216 PMC6332399

[74] Wu K Schwartz SJ Platz EA Clinton SK Erdman JW Jr Ferruzzi MG Variations in plasma lycopene and specific isomers over time in a cohort of us men. *J Nutr.* (2003) 133:1930–6. 10.1093/jn/133.6.1930 12771341

[75] Updike AA Schwartz SJ. Thermal processing of vegetables increases cis isomers of lutein and zeaxanthin. *J Agric Food Chem.* (2003) 51:6184–90. 10.1021/jf030350f 14518942

[76] Polar-Cabrera K Huo T Schwartz SJ Failla ML. Digestive stability and transport of norbixin, a 24-carbon carotenoid, across monolayers of caco-2 cells. *J Agric Food Chem.* (2010) 58:5789–94. 10.1021/jf100632t 20408560 PMC3849817

[77] Yonekura L Nagao A. Intestinal absorption of dietary carotenoids. *Mol Nutr Food Res.* (2007) 51:107–15. 10.1002/mnfr.200600145 17195263

[78] Chitchumroonchokchai C Diretto G Parisi B Giuliano G Failla ML. Potential of golden potatoes to improve vitamin A and vitamin E status in developing countries. *PLoS One.* (2017) 12:e0187102. 10.1371/journal.pone.0187102 29117188 PMC5678870

[79] Lautenschläger M Sendker J Hüwel S Galla H Brandt S Düfer M Intestinal formation of trans-crocetin from saffron extract (*Crocus sativus* L.) and in vitro permeation through intestinal and blood brain barrier. *Phytomedicine.* (2015) 22:36–44. 10.1016/j.phymed.2014.10.009 25636868

[80] Asai A Nakano T Takahashi M Nagao A. Orally administered crocetin and crocins are absorbed into blood plasma as crocetin and its glucuronide conjugates in mice. *J Agric Food Chem.* (2005) 53:7302–6. 10.1021/jf0509355 16131146

[81] Shahidi F. *Handbook of Antioxidants for Food Preservation.* Sawston: Woodhead Publishing (2015). 10.1016/B978-1-78242-089-7.00001-4

[82] Fogelman E Oren-Shamir M Hirschberg J Mandolino G Parisi B Ovadia R Nutritional value of potato (*Solanum tuberosum*) in hot climates: anthocyanins, carotenoids, and steroidal glycoalkaloids. *Planta.* (2019) 249:1143–55. 10.1007/s00425-018-03078-y 30603793

[83] Lachman J Hamouz K Orsák M Kotíková Z. Carotenoids in potatoes–a short overview. *Plant Soil Environ.* (2016) 62:474–81. 10.17221/459/2016-PSE

[84] Anstis P Northcote D. Development of chloroplasts from amyloplasts in potato tuber discs. *New Phytol.* (1973) 72:449–63. 10.1111/j.1469-8137.1973.tb04394.x

[85] Morris WL Ducreux LJ Fraser PD Millam S Taylor MA. Engineering ketocarotenoid biosynthesis in potato tubers. *Metab Eng.* (2006) 8:253–63. 10.1016/j.ymben.2006.01.001 16542864

[86] Diretto G Al-Babili S Tavazza R Scossa F Papacchioli V Migliore M Transcriptional-metabolic networks in β-carotene-enriched potato tubers: the long and winding road to the golden phenotype. *Plant Physiol.* (2010) 154:899–912. 10.1104/pp.110.159368 20671108 PMC2949014

[87] Li L Yang Y Xu Q Owsiany K Welsch R Chitchumroonchokchai C The or gene enhances carotenoid accumulation and stability during post-harvest storage of potato tubers. *Mol Plant.* (2012) 5:339–52. 10.1093/mp/ssr099 22155949

[88] Pasare S Wright K Campbell R Morris W Ducreux L Chapman S The sub-cellular localisation of the potato (*Solanum tuberosum* L.) carotenoid biosynthetic enzymes, Crtrb2 and Psy2. *Protoplasma.* (2013) 250:1381–92. 10.1007/s00709-013-0521-z 23794103

[89] Ducreux LJ Morris WL Prosser IM Morris JA Beale MH Wright F Expression profiling of potato germplasm differentiated in quality traits leads to the identification of candidate flavour and texture genes. *J Exp Bot.* (2008) 59:4219–31. 10.1093/jxb/ern264 18987392 PMC2639024

[90] Ilg A Yu Q Schaub P Beyer P Al-Babili S. Overexpression of the rice carotenoid cleavage dioxygenase 1 gene in golden rice endosperm suggests apocarotenoids as substrates in planta. *Planta.* (2010) 232:691–9. 10.1007/s00425-010-1205-y 20549230

[91] Zhou X McQuinn R Fei Z Wolters AMA Van Eck J Brown C Regulatory control of high levels of carotenoid accumulation in potato tubers. *Plant Cell Environ.* (2011) 34:1020–30. 10.1111/j.1365-3040.2011.02301.x 21388418

[92] Bruno M Beyer P Al-Babili S. The potato carotenoid cleavage dioxygenase 4 catalyzes a single cleavage of B-ionone ring-containing carotenes and non-epoxidated xanthophylls. *Arch Biochem Biophys.* (2015) 572:126–33. 10.1016/j.abb.2015.02.011 25703194

[93] Farahmand SK Samini F Samini M Samarghandian S. Safranal ameliorates antioxidant enzymes and suppresses lipid peroxidation and nitric oxide formation in aged male rat liver. *Biogerontology.* (2013) 14:63–71. 10.1007/s10522-012-9409-0 23179288

[94] Hosseinzadeh H Sadeghnia HR Rahimi A. Effect of safranal on extracellular hippocampal levels of glutamate and aspartate during kainic acid treatment in anesthetized rats. *Planta Med.* (2008) 74:1441–5. 10.1055/s-2008-1081335 18816431

[95] Puglia C Santonocito D Musumeci T Cardile V Graziano ACE Salerno L Nanotechnological approach to increase the antioxidant and cytotoxic efficacy of crocin and crocetin. *Planta Med.* (2019) 85:258–65. 10.1055/a-0732-5757 30206907

[96] Schieber A Saldaña MD. Potato peels: a source of nutritionally and pharmacologically interesting compounds-a review. *Glob Sci Book.* (2009) 3:23–9.

[97] Brown C. Antioxidants in potato. *Am J Potato Res.* (2005) 82:163–72. 10.1007/BF02853654

[98] Gregory MJ Menary RC Davies NW. Effect of drying temperature and air flow on the production and retention of secondary metabolites in saffron. *J Agric Food Chem.* (2005) 53:5969–75. 10.1021/jf047989j 16028982

[99] Moratalla-López N Bagur MJ Lorenzo C Martínez-Navarro M Salinas MR Alonso GL. Bioactivity and bioavailability of the major metabolites of *Crocus sativus* L. flower. *Molecules.* (2019) 24:2827. 10.3390/molecules24152827 31382514 PMC6696252

[100] Xi L Qian Z Xu G Zheng S Sun S Wen N Beneficial impact of crocetin, a carotenoid from saffron, on insulin sensitivity in fructose-fed rats. *J Nutr Biochem.* (2007) 18:64–72. 10.1016/j.jnutbio.2006.03.010 16713230

[101] Kyriakoudi A O’Callaghan YC Galvin K Tsimidou MZ O’Brien NM. Cellular transport and bioactivity of a major saffron apocarotenoid, picrocrocin (4-(B-D-glucopyranosyloxy)-2, 6, 6-trimethyl-1-cyclohexene-1-carboxaldehyde). *J Agric Food Chem.* (2015) 63:8662–8. 10.1021/acs.jafc.5b03363 26340688

