# Supplementary material for: Fortification and bioaccessibility of saffron apocarotenoids in potato tubers
Source: Front Nutr. 2022 Nov 30;9:1045979. doi: 10.3389/fnut.2022.1045979 (PMC9748089; doi:10.3389/fnut.2022.1045979)
Supplement: Supplementary file 1 [file Data_Sheet_1.docx]

Supplementary Material

# Supplementary Figures

Zeaxanthin

**7**

**8**

**8’**

**7’**

β-carotene

δ-carotene

α-carotene

Lutein

HO

OH

All-*trans*-lycopene

pro-lycopene

**7'**

15-*cis*-phytoene

OPP

ppo

Geranylgeranyl diphosphate

(GGPP)

**9'**

**9'**

**9**

9,9’-di-*cis*-ζ-carotene

**7**

**9**

**PSY**

**PDS**

**ZISO**

**ZDS**

**CrtISO**

**BCH**

**LCYε**

**LCYβ**

**CsCCD2L**

Crocetindial

Crocetin

Crocins

**ALDH**

**UGT**

picrocrocin

O

O-GLU

2 X 3-OH-β-Cyclocitral

O

OH

OHC

safranal

**Supplementary Figure 1.** Biosynthetic pathway of crocins

#
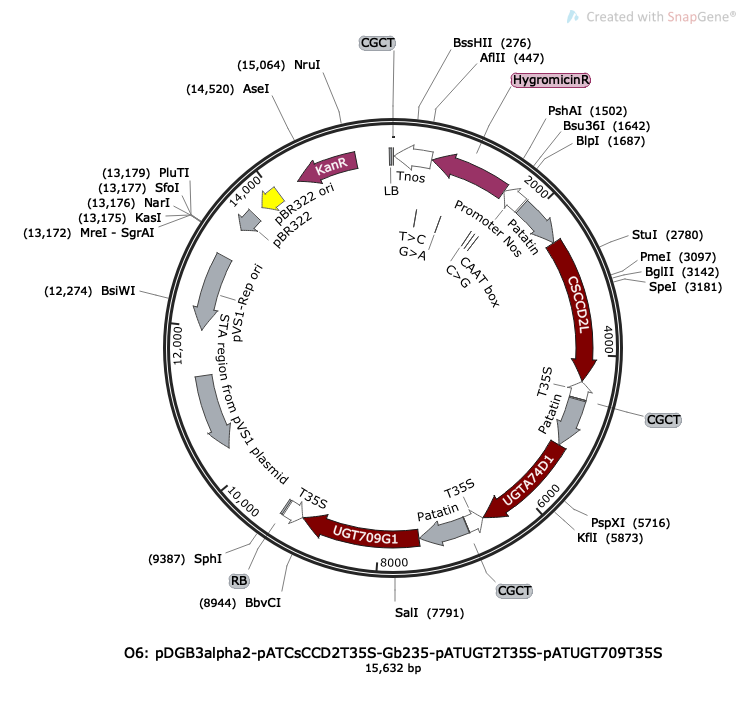


# Supplementary Figure 2. Schematic representation of the O6 construct used to transform potatoes plants cv Désirée.

# Supplementary Tables

**Supplementary Table S1.** Oligonucleotides used for plasmid construction.

| **Primers** | **Sequence 5 ́-3 ́** |
| --- | --- |
| pUPD2-Dom-UGT2-F | GCGCCGTCTCGCTCGAATGTTGAACGGCAACAAATGC |
| pUPD2-Dom-UGT2-R | GCGCCGTCTCGCTCAAAGCTTAAACTAAGGAAATTTTGGAGTCAT |
| pUPD2-Dom-CsCCD2-L-F | GCGCCGTCTCGCTCGAATGGAATCTCCTGCTACTAAATTA |
| pUPD2-Dom-CsCCD2-L-R | GCGCCGTCTCGCTCAAAGCTCATGTCTCTGCTTGGTGCT |
| pUPD2-Dom-UGT709-F | GCGCCGTCTCGCTCGAATGGCTGAGAAAGAAGCAAATAC |
| pUPD2-Dom-UGT709-R | GCGCCGTCTCGCTCAAAGCTCAGGTCCGAAGAAAATTTGG |
| pUPD2-Dom-pPAT-F | GCGCCGTCTCGCTCGGGAGAATTTGTCAAATCAGGCTCAAAGA |
| pUPD2-Dom-pPAT-R | GCGCCGTCTCGCTCACATTCTTTGCAAATGTTCAAAGTGTTTTTA |

**Supplementary Table S2.** Oligonucleotides used for expression analyses.

| Gene name | Oligonucleotide forward5ʹ-3ʹ | Oligonucleotide reverse5ʹ-3ʹ | Gene/reference |
| --- | --- | --- | --- |
| CsUGT2 | TCGAGCCTAGTGATCTGCCGT | TCGAGCCAGTCCAAGTAGGGA | AY262037 |
| UGT709G1 | ACTCACCTCCACGTCCTTCCAG | CTTGATCAGCCACGACACAAG | KX385186.1 |
| CsCCD2L | ACATGTCGCCTTGAGAGTCC | TCAGATTTGATGCCAGGTTG | KP887110 |
| CCD1 | CGAGAACTACACTGGCAGGA | CTCCGAACCAAATCTGCCAG | XM_006345381 |
| CCD4 | ACCCGCCCATCATTTCTACA | CTCATCCTTGGCGTAACGTG | XM_006359904.2 |
| Actin | GCTTCCCGATGGTCAAGTCA | GGATTCCAGCTGCTTCCATTC | X55749 |
| DXS | GCCGCCATTGATGACAGACCA | TCCCCCTCAATCAATATCCTACCT | AF143812 |
| DXR | TGGCCCTCAATTTGCTTTCTCCTA | CCCCACTGCACCCCTTTCTTCT | BE924542 |
| BCH1 | CTTGGCCCAAAACCCACTT | CCTCAAATTGAGGTTTCAGCTTCT | (51) |
| BCH2 | TTTTGCTGTCTCGAAGAAAGCC | AGCCAACAGGCAGCTAAACTCT | (51) |
| PSY1 | CGCAAGATACTGGACGAGATT | TTTGCTAGTGGGGAAGAAGTTGAC | TC122598 |
| PSY2 | AGCTTTAGATAGGTGGGAGGCA | CAAGTCCATACGCATTCCTTCAA | (51) |
| PDS | AATCACCGAAAGCAGGCATCT | AATTTGTGGTGGTTTGGCAGTTAC | TC28515 |
| ZDS | TATTCGGGCTGATTTGGACTCT | AGCTTTGGCCCCCGATAA | BG592944 |
| CrtISO | TTGGCAGCAGTAGGACGTAAAC | TCCCTTCCTTTTCATGTGGAA | TC117194 |
| LCY-e | TGGCCACAAGAACGAAAACGAC | GCGCGGAAAAATGACCTTATC | AF321537 |
| LCY-b1 | TTATGGCATTTTGGCTGAAGTG | TGGCATTGCATAAAGAAAAGTTG | X86452 |
| LYC-b2 | AATGGGTGGTCCACTTCCAGTA | GGATGGATGAACCATGCCAG | (51) |
| ZISO | TCTGCTGAATTTGAGTTATCC | GCAATGCTGTTTAATTGCCA | XM_006349824 |
